# Multinational Analysis of Estimated Health Care Costs Related to Extended-Interval Fixed Dosing of Checkpoint Inhibitors

**DOI:** 10.1001/jamanetworkopen.2023.0490

**Published:** 2023-02-23

**Authors:** Daniel A. Goldstein, Gary M. Ginsberg, Dorit Hofnung-Gabbay, Richard De Abreu Lourenco, Herbert H. Loong, Boon Cher Goh, Kelvin K. W. Chan, Massimo Di Maio, Francesco Perrone, Peter S. Hall, Alona Zer, Eli Rosenbaum

**Affiliations:** 1 Faculty of Medicine, Tel Aviv University, Tel Aviv, Israel; 2 Davidoff Cancer Center, Rabin Medical Center, Petah Tikva, Israel; 3 Department of Health Policy and Management, Gillings School of Public Health, University of North Carolina, Chapel Hill; 4 Clalit Health Service, Tel Aviv, Israel; 5 Hebrew University–Hadassah Braun School of Public Health, Jerusalem, Israel; 6 Centre for Health Economics Research and Evaluation, University of Technology Sydney, Ultimo, Australia; 7 Department of Clinical Oncology, The Chinese University of Hong Kong, Hong Kong, China; 8 Department of Hematology-Oncology, National University Cancer Institute, Singapore; 9 Sunnybrook Odette Cancer Centre, University of Toronto, Toronto, Canada; 10 Canadian Centre for Applied Research in Cancer Control, Toronto, Canada; 11 Department of Oncology, University of Turin, Ordine Mauriziano Hospital, Turin, Italy; 12 Clinical Trial Unit, Istituto Nazionale Tumori, IRCCS Fondazione Pascale, Napoli, Italy; 13 Edinburgh Cancer Research Centre, University of Edinburgh, Edinburgh, United Kingdom; 14 Institute of Oncology, Rambam Medical Center, Haifa, Israel

## Abstract

**Importance:**

New dosing options for immune checkpoint inhibitors have recently been approved by the US Food and Drug Administration (FDA), including fixed dosing with extended intervals. Although the dose intensity appears the same, there is expected to be some waste with extended-interval dosing, as some drug remains in the bloodstream once a decision to stop treatment is made. The economic impact of extended-interval fixed dosing is unknown compared with standard-interval fixed dosing.

**Objective:**

To analyze the potential health care costs of using extended-interval fixed dosing instead of standard-interval fixed dosing.

**Design, Setting, and Participants:**

This economic evaluation used a pharmacoeconomic model to simulate 2 cohorts of patients with platinum-resistant metastatic urothelial cancer receiving pembrolizumab as second-line therapy at different dosing intervals using 2020 pricing data. Data were analyzed from 2020 to 2022.

**Exposures:**

The simulated patients received FDA-approved regimens of either 200 mg every 3 weeks or 400 mg every 6 weeks.

**Main Outcomes and Measures:**

The progression-free survival curve from the KEYNOTE-045 trial was used to estimate treatment duration. Drug, imaging, and administration costs were included in analyses. Sensitivity analyses were performed to assess how different imaging frequencies would affect the model results. The potential overall costs of using the 2 different dosing strategies were assessed. The base case was set in the US, while sensitivity analyses were set in several other countries.

**Results:**

In the base case analysis, dosing every 6 weeks instead of every 3 weeks resulted in an estimated 8.9% increase in pembrolizumab costs for the health care payer. Accounting for a decrease in infusion costs would result in an estimated net additional cost of $7483 per patient in the US (7.9% cost increase). In the US, this would amount to an increase of approximately $28 million annually for health care payers. Similar percentages in cost estimate increases were found for health care payers around the world, such as in Israel, where the net additional cost would be $5491 per patient.

**Conclusions and Relevance:**

This economic evaluation assessed and quantified the potential increased costs related to extended-interval fixed dosing of pembrolizumab. The model method could be applied to other diseases and other drugs for which there has been a movement toward extended-interval dosing. Results may differ in other diseases owing to differing disease courses and patient profiles.

## Key Points

**Question:**

Is extended-interval fixed dosing of pembrolizumab associated with increased health care costs?

**Findings:**

This economic evaluation including simulated patients found that using the US Food and Drug Administration–labeled pembrolizumab dose of 400 mg every 6 weeks instead of 200 mg every 3 weeks may result in an estimated 8% increase in health care costs in the US.

**Meaning:**

These findings suggest that when considering different dosing options, clinicians and policy makers should consider the potential impact on costs.

## Teaser

This economic evaluation estimates the health care costs associated with using extended-interval fixed dosing instead of standard-interval fixed dosing of immune checkpoint inhibitors in a simulated cohort of patients with urothelial cancer.

## Introduction

As of January 2021, pembrolizumab has received 39 US Food and Drug Administration (FDA) approvals for the treatment of cancer. Initially approved with weight-based dosing, there was a subsequent movement toward using fixed doses for all patients, first with 200 mg every 3 weeks, and more recently 400 mg every 6 weeks.^1^ It has previously been argued that the move to fixed dosing from weight-based dosing has maintained efficacy but increased costs.^2^ While lower doses of immune checkpoint inhibitors have been strongly recommended in other publications,^3,4,5^ we recognize that the FDA-approved dose levels and schedules remain the standard of care in many health care settings.

As the dose intensity and price of pembrolizumab have essentially remained constant, it has been suggested that the cost of 400 mg every 6 weeks is equivalent to 200 mg every 3 weeks. We hypothesized that 400 mg every 6 weeks in fact represents a cost increase for health care payers. In clinical practice, when a patient’s disease progresses, the treatment is usually stopped irrespective of when the last treatment was received. However, if a patient is treated with larger, but less frequent doses, some of the drug may remain in the bloodstream, which would not otherwise have been infused if they had been receiving more frequent treatment at lower doses. The frequency of imaging studies to determine progression status will affect the costs associated with the duration of treatment. It is crucial to emphasize that a large body of literature exists to highlight that there are no expected differences in efficacy or safety of these 2 dosing strategies.^6^

We hypothesized that 400 mg every 6 weeks would be costlier for health care payers than 200 mg every 3 weeks for patients receiving pembrolizumab as second-line treatment for metastatic urothelial cancer. The primary aim of this study was to estimate whether extended interval dosing was more costly and, if so, to quantify this cost increase. The secondary aim was to assess the influence on the results of different drug and health care costs around the world. To examine this hypothesis, we used a cost minimization analysis to simulate the number of infusions, imaging studies, and drug consumption for various imaging frequency protocols for patients with metastatic urothelial cancer. We chose urothelial cancer because at the time of model development, pembrolizumab was a commonly accepted standard of care, and full survival data have been published.

## Methods

This economic evaluation used a pharmacoeconomic model without the use of individual patient data; therefore, the study was exempt from the need for ethical approval or informed consent. This study follows the Consolidated Health Economic Evaluation Reporting Standards (CHEERS) reporting guideline.

### Statistical Analysis

#### Overall Model Structure

We used an Excel-based mathematical model (Microsoft) to simulate a population of patients receiving pembrolizumab as second-line treatment of metastatic urothelial cancer. The model ran from 0 to 104 weeks. Treatment was stopped at 2 years (104 weeks), in accordance with the registration trial protocol design, and common reimbursement restrictions. We estimated the duration of treatment until the medical decision to stop treatment. We incorporated data on drug and infusion costs. Our focus was to estimate the difference in treatment costs associated with the 2 dosing strategies for fixed dosing pembrolizumab: 200 mg every 3 weeks vs 400 mg every 6 weeks.

#### Duration of Treatment

We simulated individual patients and extracted the progression-free survival (PFS) data of the pembrolizumab group from the registration clinical trial (KEYNOTE-045) to simulate the duration of treatment for each patient.^7^ For scenarios in which computed tomography (CT) imaging was performed in the same week as in the clinical trial, we used the trial’s PFS data. Since the largest decreases in the PFS data were only seen at the time of imaging, we interpolated these decreases by assuming that there was an underlying equal gradual PFS trend between each imaging event. These underlying PFS trend data were used for scenarios when CT imaging was performed in a week that had not been performed in the clinical trial. Further details of the modeling methods are included in the eMethods in Supplement 1.

The median duration of treatment in the KEYNOTE-045 trial was 3.5 months, and the median duration of treatment in our model was 3.3 months, closely replicating the median PFS in KEYNOTE-045. As in KEYNOTE-045, our model included a group of patients with durable responses to pembrolizumab. In KEYNOTE-045, the 12-month PFS was 17%, and this was exactly replicated in the model.

#### Treatment Costs

To calculate treatment costs incurred in the US, we used a commonly used method described by Tumeh et al.^8^ This method incorporates all direct costs from the hospital to the Centers for Medicare & Medicaid Services (CMS). Although there may be some variation in fees to private insurers, given that treatment for most patients with cancer is funded by CMS, we believe that this provides a close approximation. We used the June 2020 average sales price of $50.02 per mg of pembrolizumab.^9^ Treatment costs of $142.55 were based on a 1-hour infusion (Common Procedural Terminology [CPT] code 96413) according to the 2020 Medicare physician fee schedule.^10^ CT scan costs of $492.99 were based on the sum of an abdomen and pelvic scan with contrast (CPT code 74177) and a chest CT scan without contrast (CPT code 71250). As there was not expected to be any difference in adverse events rates (AEs) between the 2 groups of the model, this model did not consider the cost of AEs. Costs and consequences were evaluated over the full 104-week time horizon of the study to capture all the effects of differential dosing strategies, which ceased per the trial protocol after 104 weeks.

#### International Comparisons

We performed cross-country comparisons using country-specific input values for drug and infusion costs. Recognizing that drug efficacy was expected to be constant across countries, we assumed that the duration of treatment would also be constant across countries. Country-specific drug and infusion costs were based on publicly listed prices, which did not incorporate any additional confidential negotiated discounts between the manufacturers and health care systems. Local currencies were converted to US dollars using the exchange rates as of mid-2020.

#### Scenarios With Different Radiographic Imaging Intervals

There are different clinical approaches to the frequency of required imaging, which may impact the time point of clinical decision-making and thus the duration of therapy. While some clinicians may perform imaging at different intervals, such as every 9 or 12 weeks, there may be other significant variations in imaging frequency in the clinical setting. This may be partially driven by payer approval policies or imaging capacity constraints. We therefore performed separate simulations to assess the potential impact of 3 different imaging strategies on overall costs in the model.

In the base case imaging scenario, we assumed that imaging started at week 9 and was repeated every 9 weeks thereafter, up to and including week 99 (ie, imaging at weeks 9, 18, 27, and so on). In the second imaging scenario, we assumed that imaging started at week 12 and was repeated every 12 weeks thereafter, up to and including week 96 (ie, at weeks 12, 24, 36, and so on). In the third imaging scenario, we assumed that imaging started at week 9 and was repeated every 6 weeks for the first year and subsequently every 12 weeks, up to and including week 99 (ie, at weeks 9, 15, 21, 27, 33, 39, 45, 51, 63, 75, 87, and 99).

Based on informal surveys of medical oncologists in different health care settings around the world, we believe that imaging scenario 1 is a common option in clinical practice; however, we recognize that scenario 2 may also be common. Scenario 3 was included because this was the imaging protocol used in the KEYNOTE-045 trial.^7^ Performing imaging every 12 weeks in scenario 2 will always be in sync with 6-week treatments, thus providing a low estimate of drug waste. By contrast, in scenario 3, imaging will always be out of sync with 6-week treatments, thus providing a high estimate of waste. In scenario 1, imaging will alternate between being in sync and out of sync with 6-week treatments, thus providing an estimate between the estimates of scenarios 2 and 3. In essence, when imaging and infusions are in sync, there should be no waste, and when they are completely out of sync, it enables us to calculate the maximum possible waste.

#### Sensitivity Analysis

##### Mixed Dosing Strategies

We hypothesized that dosing every 3 weeks would be cheaper in terms of drug costs alone than dosing every 6 weeks. However, recognizing that infusion costs will be higher for the more frequent treatment strategy, we suspected that there may be a transition point for patients with durable responses at which the less frequent dosing becomes less costly due to lower administration costs. We sought to identify whether and when this transition point occurs. Therefore, assuming imaging scenario 1, further sensitivity analyses were performed in which infusions were initially given every 3 weeks but transitioned to every 6 weeks at week 30, 42, 54, or 84.

##### Weight-Based Dosing

Some health care systems around the world use a weight-based dosing approach, using 2 mg/kg every 3 weeks or 4 mg/kg every 6 weeks.^11^ Given that the mean weight of a patient with cancer is 75 kg, we performed a sensitivity analysis using mean doses of 150 mg every 3 weeks compared with 300 mg every 6 weeks.

#### Health Care Payer vs Manufacturer Perspectives

While we recognize that for the health care payer, the differences in infusion costs are relevant, they are not relevant for the manufacturer. We therefore assessed the overall costs and revenue from both of these different perspectives.

#### Overall Budget Impact

Based on first quarter reports in 2020, global pembrolizumab sales in 2020 were estimated to be approximately $13.1 billion.^12^ Approximately 56% of sales ($7.3 billion) were in the US.^13^ According to the manufacturer, urothelial cancer accounted for 5% of all sales.^14^ Therefore, 2020 expenditure on pembrolizumab in the US is estimated to have been approximately $367 million. We sought to estimate the overall budget impact of the 3- and 6-week dosing strategies in the US under different imaging scenarios, modified by the application of an annual discount rate of 3%^15^ applied to all costs incurred during the second year of the evaluation. Data were analyzed from 2020 to 2022.

## Results

**Figure 1.**
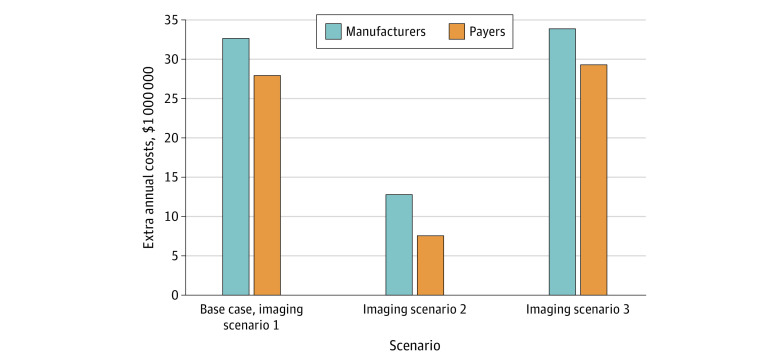
Estimated Extra Annual Costs of 6-Week Dosing Schedule in the US From Manufacturers and Payers’ Perspectives

**Figure 2.**
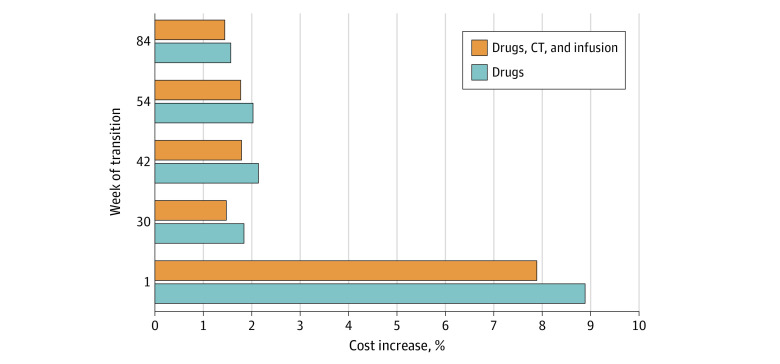
Estimated Costs of Mixed Dosing Strategy Using Base Case Imaging Scenario 1 Week of transition is when patients are transitioned from a 3-week to 6-week dosing schedule.

In the base case imaging scenario 1, we estimate that dosing every 6 weeks instead of every 3 weeks would result in an 8.9% increase in pembrolizumab costs for the health care payer (Table 1, Figure 1). This strategy would result in an annual increase in manufacturers revenues in the US of approximately $33 million (Figure 1). From the health care payer’s perspective, the increase in drug costs would be partially offset by a decrease in infusion and imaging costs, resulting in a 7.9% net cost increase (eTable 1 in Supplement 1), or approximately $28 million (Figure 1). Figure 2 illustrates mixed dosing strategies. If the transition to the 6-week dosing schedule occurred at week 30, total cost increases would be reduced to 1.5%, and transition at week 42 would result in an increase of 1.8% (eTable 2 in Supplement 1). No additional significant cost differences would occur if the transition occurred at 54 or 84 weeks.

**Table 1.** Estimated Additional Drug Costs in the US of Substituting a 6-Week Dosing Protocol Instead of the 3-Week Protocol

Imaging frequencies	Infusions per patient, mean, No.		Annual drug costs of dosing, $		Drug cost increase, %	Annual drug cost increase, $
	200 mg every 3 wk	400 mg every 6 wk	Every 3 wk	Every 6 wk		
Every 9 wk	9.142	4.977	367 151 200^a^	399 800 693	8.9	32 649 493
Every 12 wk	9.567	4.942	384 219 801	396 973 401	3.3	12 753 601
Every 6 wk then 12 wk	9.022	4.932	362 304 892	396 212 718	9.4	33 907 825

^a^ Calculations assumed that the US accounts for 55.9% of global sales, of which 5% (ie, 2.8% of global sales) are associated with treating urothelial cancer.

In imaging scenario 2, we estimate that dosing every 6 weeks instead of every 3 weeks would result in a 3.3% increase in pembrolizumab costs for the health care payer (Table 1, Figure 1). This strategy would result in an annual increase in manufacturer revenues in the US of approximately $13 million (Figure 1). From the health care payer’s perspective, the increase in drug costs would be partially offset by a decrease in infusion and imaging costs, resulting in a 2.5% net cost increase (eTable 1 in Supplement 1), or $7.5 million (Figure 1).

In imaging scenario 3, we estimate that dosing every 6 weeks instead of every 3 weeks would result in a 9.4% increase in pembrolizumab costs for the health care payer (Table 1, Figure 1). This strategy would result in an annual increase in manufacturers revenues in the US of approximately $34 million (Figure 1). From the health care payer’s perspective, the increase in drug costs would be partially offset by a decrease in infusion and imaging costs, resulting in an 8.3% net cost increase (eTable 1 in Supplement 1), or $29 million (Figure 1).

**Figure 3.**
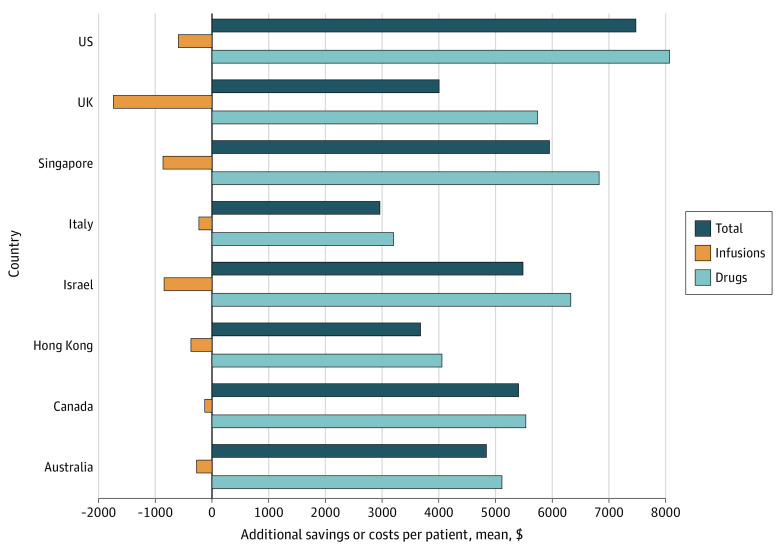
Estimated Mean Additional Costs Using 6-Week Dosing Schedule Instead of 3-Week Dosing Schedule, Per Patient and by Country Costs were modeled with baseline imaging scenario 1.

Table 2 and Figure 3 show unit drug and infusion costs for selected countries. As a result of moving from a 3-week to 6-week dosing protocol, additional drug costs per patient would range from $3205 (in Italy) to the aforementioned $8072 in the US. After considering the cost savings resulting from fewer infusions, the net cost increase for health care payers range from $2965 in Italy to $7483 in the US (Table 2). In an additional sensitivity analysis on the base case using mean weight-based dosing, we estimate that 300 mg every 6 weeks instead of 150 mg every 3 weeks would result in an 8.5% net cost increase, or $31 million, from the US payers’ perspective.

**Table 2.** Estimated Unit Costs and Additional Costs Per Patient By Country Related to Moving to 6-Week Dosing Schedule From the Current 3-Week Protocol^a^

Country	Local currency		US$				
	Infusion cost^b^	Drug cost for 200 mg pembrolizumab^c^	Infusion cost^b^	Drug cost for 200 mg pembrolizumab^c^	Drug costs per patient^c^	Savings in infusion costs	Total extra costs
Australia	A$85.25	A$8289	66	6340	5115	−273	4843
Canada	CAD$41.86	CAD$8800	32	6873	5545	−132	5413
Hong Kong	HK$715	HK$39 000	92	5030	4058	−379	3679
Israel	₪657	₪25 258	204	7851	6334	−843	5491
Italy	€47	€3240	58	3972	3205	−240	2965
Singapore	SGD280	SGD11 221	211	8464	6829	−872	5957
United Kingdom	£312	£5260	422	7121	5745	−1744	4001
United States	$143	$10 005	143	10 005	8072	−589	7483

^a^ Calculations used the every 9 weeks imaging schedule.

^b^ The infusion cost is considered to incorporate the institutional cost of providing the infusion, which this generally incorporates pharmacy and nursing costs but does not include a physician visit.

^c^ The drug cost is considered to incorporate only the cost of the drug. However, due to different payment structures in different countries, these costs are not perfectly comparable. Drug costs are from publicly published sources. Additional confidential discounts may occur subsequently. It should also be noted, that despite some differences in how infusion costs are obtained, their overall influence on the model results is minor. Cost sources are included in eTable 3 in Supplement 1.

## Discussion

This economic evaluation estimates the potential financial impact of extended-interval dosing of cancer drugs. We used the example of pembrolizumab in metastatic urothelial cancer as an example to demonstrate this issue; however, this concept is certain to be applicable to other cancers using the same drug as well as other drugs. To our knowledge, this is the first pharmacoeconomic study to demonstrate this issue and to provide the modeling method. Furthermore, we demonstrated how the model could be used and that it was robust when applied to health care systems in different countries.

It is important to highlight that, in our model's estimates, increased pharmaceutical costs (as a result of moving to a 6-week dosing protocol) to health care payers would be only partially ameliorated by resultant savings in infusion costs. From the manufacturer’s perspective, replacing 3-week dosing with 6-week dosing led to an increase in sales revenue in all possible scenarios. While our model demonstrated potential financial impact to manufacturers and health care payers, it is fair to assume that there may be some impact on individual patients, depending on which country they live in and how their health care is financed. Clearly, there is also a benefit to 6-week dosing, namely patient convenience due to fewer visits to the hospital and potentially also less exposure to infections from hospital visits. The question for policy makers is whether the increased cost is justified by the increased convenience. Our study provides financial data, thus helping policy makers to more fully consider this trade-off.

### Limitations

This study has some limitations. As with all models, there are limitations based on estimations used in the model inputs. The precise treatment duration was estimated using PFS as a surrogate. While we believe this to be a good surrogate, it may not be perfectly accurate. Furthermore, in the KEYNOTE-045 trial, there were some patients who continued therapy beyond progression for unclear reasons. We did not incorporate this phenomenon into the model, as we suspect that this would not be common practice in the practice setting. Using the clinical trial as the basis for the model may also be problematic in some respects, as clinical trials are often not fully representative of the practice setting. There may be intracountry and intercountry differences in patterns of care due to socioeconomic diversity, which may lead to variations in treatment durations. We used cost inputs that were publicly available; however, there may be subsequent confidential discounts provided to health care payers. There may also be intracountry price differences; however, we tried to use price estimates that were most representative of the overall population in each country. In estimating drug and infusion costs, there is some variation among countries. While some variation was expected, the precise reasons for variation and its magnitude are unclear. We used second-line pembrolizumab as the example in our model; however, we recognize that the use of this regimen in this setting may decrease with time due to the recent publication of first-line switch maintenance with avelumab.^16^ While not all patients will receive maintenance avelumab, as the uptake increases, the use of second-line pembrolizumab will inevitably decrease. This will not affect the percentage increased cost of using extended interval dosing, but it will reduce the total increase in expenditure. While this model design can be replicated for other malignant neoplasms, differing drug efficacy, patterns of ongoing monitoring, and treatment durations may lead to different results. We recognize that due to the unfavorable prognosis in the setting of second-line urothelial cancer, treatment interruptions may be more common than in the setting of other diseases. A limitation of this modeling study is that it uses set imaging frequency protocols, and does not account for some clinical situations of off-schedule imaging due to onset of new symptoms. Our clinical impression is that these situations would be uncommon, would be balanced in both groups of the model, and would not have a major impact on the model results.

There are of course many other issues that affect costs when using immunotherapy in cancer care, and in this economic evaluation, we are highlighting only one of these issues. Costs could be reduced by reducing doses: while the current fixed dosing strategies appear on the FDA label, it is well established that weight-based dosing can provide the same efficacy while reducing costs.^3^ Such an approach has been incorporated by national health care agencies, such as in Canada.^11^ Furthermore, the need for prolonged therapy in responding patients is an open question. While most of the clinical trial protocols used therapy up to a maximum of 2 years, this may not be necessary.

## Conclusions

In this economic evaluation model comparing different fixed dosing options of pembrolizumab, we found an approximately 8% estimated increase in health care costs when using the FDA-labeled dose of 400 mg every 6 weeks instead of 200 mg every 3 weeks. As new treatments and technologies are developed, health care payers need to continue to be mindful of cost implications prior to implementation. This study provides a new concept and analytic model for consideration.

## Notes

Supplement 1.
eMethods.
**eTable 1.** Mean Cost Per Patient by Differing Imaging and Dosage Strategies **eTable 2.** Cost Impact of Mixed Dosing Strategies Per Patient With Imaging Every 9 Weeks Compared With Base Case of Dosing Every 3 Weeks **eTable 3.** Cost Sources

Supplement 2.
Data Sharing Statement

## References

[1] Lala M, Li M, Sinha V, de Alwis D, Chartash E, Jain L. A six-weekly (Q6W) dosing schedule for pembrolizumab based on an exposure-response (E-R) evaluation using modeling and simulation. J Clin Oncol. 2018;36(15)(suppl):3062. doi:10.1200/JCO.2018.36.15_suppl.306230188790

[2] Caglio A, Gamba T, Tagini V, . Evaluation of the economic impact of body weight (BW)-based dose (3mg/kg) versus flat dose of nivolumab: a single center experience. Tumori J. 2020;106(2)(suppl):1-215. doi:10.1177/0300891620953388

[3] Goldstein DA, Gordon N, Davidescu M, . A phamacoeconomic analysis of personalized dosing vs fixed dosing of pembrolizumab in firstline PD-L1–positive non–small cell lung cancer. J Natl Cancer Inst. 2017;109(11). doi:10.1093/jnci/djx063 29059432

[4] Ratain MJ, Goldstein DA. Time is money: optimizing the scheduling of nivolumab. J Clin Oncol. 2018;36(31):3074-3076. doi:10.1200/JCO.18.00045 30148658

[5] Goldstein DA, Ratain MJ. Alternative dosing regimens for atezolizumab: right dose, wrong frequency. Cancer Chemother Pharmacol. 2019;84(6):1153-1155. doi:10.1007/s00280-019-03971-7 31630223

[6] US Food and Drug Administration. FDA approves new dosing regimen for pembrolizumab. Accessed April 9, 2022. https://www.fda.gov/drugs/resources-information-approved-drugs/fda-approves-new-dosing-regimen-pembrolizumab

[7] Bellmunt J, de Wit R, Vaughn DJ, ; KEYNOTE-045 Investigators. Pembrolizumab as second-line therapy for advanced urothelial carcinoma. N Engl J Med. 2017;376(11):1015-1026. doi:10.1056/NEJMoa1613683 28212060 PMC5635424

[8] Tumeh JW, Moore SG, Shapiro R, Flowers CR. Practical approach for using Medicare data to estimate costs for cost-effectiveness analysis. Expert Rev Pharmacoecon Outcomes Res. 2005;5(2):153-162. doi:10.1586/14737167.5.2.153 19807571

[9] Centers for Medicare & Medicaid Services. 2020 ASP drug pricing files. Accessed June 17, 2020. https://www.cms.gov/medicare/medicare-part-b-drug-average-sales-price/2020-asp-drug-pricing-files

[10] Centers for Medicare & Medicaid Services. Physician fee schedule. Accessed June 17, 2020. https://www.cms.gov/apps/physician-fee-schedule/search/search-criteria.aspx

[11] Walker S, de Léséleuc L, Butcher R. CADTH Technology Review: Optimal Use 360 Report Dosing and Timing of Immuno-Oncology Drugs. Canadian Agency of Drugs and Technologies in Health; 2019.

[12] Merck. Merck announces first quarter 2020 results. Accessed July 8, 2020. https://www.merck.com/news/merck-announces-first-quarter-2020-financial-results/

[13] DrugAnalyst. Drug Analyst database. Accessed July 8, 2020. https://www.druganalyst.com/mrk/keytruda

[14] MRK—Q1 2019 Merck & Co Inc earnings call. Transcript. *Thomson Reuters Street Events*. April 30, 2019. Accessed May 6, 2020. https://s21.q4cdn.com/488056881/files/doc_financials/2019/MRK-USQ_Transcript_2019-04-30-(1).pdf

[15] Haacker M, Hallett TB, Atun R. On discount rates for economic evaluations in global health. Health Policy Plan. 2020;35(1):107-114. doi:10.1093/heapol/czz127 31625564

[16] Powles T, Park SH, Voog E, . Avelumab maintenance therapy for advanced or metastatic urothelial carcinoma. N Engl J Med. 2020;383(13):1218-1230. doi:10.1056/NEJMoa2002788 32945632

