# Supplementary material for: Multinational Analysis of Estimated Health Care Costs Related to Extended-Interval Fixed Dosing of Checkpoint Inhibitors
Source: JAMA Netw Open. 2023 Feb 23;6(2):e230490. doi: 10.1001/jamanetworkopen.2023.0490 (PMC9951041; doi:10.1001/jamanetworkopen.2023.0490)
Supplement: Supplement 1. — eMethods. eTable 1. Mean Cost Per Patient by Differing Imaging and Dosage Strategies eTable 2. Cost Impact of Mixed Dosing Strategies Per Patient With Imaging Every 9 Weeks Compared With Base Case of Dosing Every 3 Weeks eTable 3. Cost Sources [file jamanetwopen-e230490-s001.pdf]

Supplemental Online Content

Goldstein DA, Ginsberg GM, Hofnung-Gabbay D, et al. Multinational analysis of estimated health care costs related to extended-interval fixed dosing of checkpoint inhibitors. *JAMA Netw Open*. 2023;6(2):e230490.
doi:10.1001/jamanetworkopen.2023.0490

eMethods.
eTable 1. Mean Cost Per Patient by Differing Imaging and Dosage Strategies
eTable 2. Cost Impact of Mixed Dosing Strategies Per Patient With Imaging Every 9 Weeks Compared With Base Case of Dosing Every 3 Weeks
eTable 3. Cost Sources

This supplemental material has been provided by the authors to give readers additional information about their work.

© 2023 Goldstein DA et al. *JAMA Network Open.*

eMethods.

The modelling process to estimate the number of people who were dosed with Pembrolizumab in each scenario were as follows:-

For Scenario 1 (CT every 9 weeks)

A) Prior to the first CT (ie: at weeks 3 and 6 we used the overall survival (OS) data as no a-priori information existed as to who were progression free.
B) At week 9 when the first CT was performed, the timing coincided with the first trial CT (Scenario 3: CT at weeks 9,15,21,27,33,39,45,51,63,75,87,99) so we used the progression free survival (PFS) trial data.
C) At week 12, we used the trials PFS data, that took into account any deaths that had occurred since the trials last CT at week 9.
D) At week 15, unlike in the trial, no CT was performed. Therefore, we adjusted the PFS data at week 12, by the OS curve in order to estimate how many PFS at week 12 were alive at week 15 and therefore eligible for dosing.
E) At week 18 (when the second CT was performed), we interpolated the trial PFS data from the CTs performed at weeks 15 and 21, to estimate the underlying PFS at week 18.
F) At weeks 21 and 24, we adjusted the PFS at week 18 by the appropriate OS curve to estimate how the number of PFS survivors who were eligible for dosing.
G) At week 27 when the third CT was performed, the timing coincided with the fourth trial CT so we used the progression free survival (PFS) trial data.

For Scenario 2 (CT every 12 weeks)

A) Prior to the first CT (ie: at weeks 3, 6 and 9 we used the overall survival (OS) data as no a-priori information existed as to who were progression free.
B) At week 12 (when the first CT was performed), we interpolated the trial PFS data from the CTs performed at weeks 9 and 15, to estimate the underlying PFS.
C) At weeks 15, 18 and 21 no CT was performed. Therefore, we adjusted the PFS data at week 12, by the appropriate OS curve in order to estimate how many confirmed PFS at week 12 were alive and therefore eligible for dosing.
D) At week 24 (when the second CT was performed), we interpolated the trial PFS data from the CTs performed at weeks 21 and 27, to estimate the underlying PFS at week 24.
E) At weeks 27, 30 and 33 no CT was performed. Therefore, we adjusted the PFS data at week 24, by the appropriate OS curve in order to estimate how many confirmed PFS were alive and therefore eligible for dosing.
F) At week 36 (when the third CT was performed), we interpolated the trial PFS data from the CTs performed at weeks 33 and 39, to estimate the underlying PFS at week 36. Etc…..

For Scenario 3

Since this was the trial scenario, the trial PFS were used throughout.

© 2023 Goldstein DA et al. *JAMA Network Open.*

eTable 1. Mean Cost Per Patient by Differing Imaging and Dosage Strategies

	Average number of infusions per patient	Drug costs	CT & Infusion costs	CT & Drug & infusions	Extra % cost
Base case: imaging scenario 1					
(every nine weeks)					
dosing every three weeks	9.142	$90,771	$4,005	$94,777	
dosing every six weeks	4.977	$98,843	$3,416	$102,259	7.90%
Cost difference		$8,072	-$589	$7,483	
Imaging scenario 2					
(every 12 weeks)					
dosing every three weeks	9.567	$95,021	$3,433	$98,453	
dosing every six weeks	4.942	$98,144	$2,778	$100,923	2.51%
Cost difference		$3,124	-$655	$2,469	
Imaging scenario 2					
(every 6 then 12 weeks)					
dosing every three weeks	9.022	$89.579	$4,581	$94,160	
dosing every six weeks	4.932	$97,956	$4,003	$101,159	8.28%
Cost difference		$8,377	-$579	$7,799	

© 2023 Goldstein DA et al. *JAMA Network Open.*

eTable 2. Cost Impact of Mixed Dosing Strategies Per Patient With Imaging Every 9 Weeks Compared With Base Case of Dosing Every 3 Weeks

	**Pembrolizumab (mg)**	**No. of Infusions**	**Drug Cost**	**extra % cost**	**CT & Infusion Cost**	**Drug, CT & Infusion Costs**	**Extra % cost**
**Infusions:-**							
**Every 3 weeks (Base Case)**	1828	9.1	$90,771		$4,005	$94,777	
**Every 6 weeks**	1991	5.0	$98,843	8.89%	$3,416	$102,259	7.90%
**transition at 30**	1867	7.2	$92,445	1.84%	$3,730	$96,176	1.48%
**transition at 42**	1863	7.4	$92,709	2.13%	$3,766	$96,475	1.79%
**transition at 54**	1860	8.0	$92,609	2.02%	$3,851	$96,460	1.78%
**transition at 84**	1855	8.7	$92,198	1.57%	$3,949	$96,147	1.45%

© 2023 Goldstein DA et al. *JAMA Network Open.*

eTable 3. Cost Sources

	Infusion Cost Source	Drug Cost Source
Australia	Australian Government MBS code 14245	Australian Government Pharmaceutical Benefits Scheme
Canada	Sunnybrook Odette Cancer Centre Pharmacy	Sunnybrook Odette Cancer Centre Pharmacy
Hong Kong	Government of Hong Kong	
Israel	Israel Ministry of Health	Israel Ministry of Health
Italy	Based on the estimation of time required for preparation (pharmacy) and administration (nurses) and on the 2019 average salaries at Mauriziano Hospital, Turin	Official hammer price of pembrolizumab in the tender by Piedmont Region
Singapore	Based on an estimate that Infusion costs are approx 2.5% of drug costs in Singapore	Public academic healthcare institution pharmacy inventory
UK	National Health Service Reference Costs	British National Formulary
USA	2020 Medicare physician fee schedule code 96413	June 2020 Centers for Medicare and Medicaid Services Average Sales Price

© 2023 Goldstein DA et al. *JAMA Network Open.*
