# Supplementary material for: Multinational Analysis of Estimated Health Care Costs Related to Extended-Interval Fixed Dosing of Checkpoint Inhibitors
Source: JAMA Netw Open. 2023 Feb 23;6(2):e230490. doi: 10.1001/jamanetworkopen.2023.0490 (PMC9951041; doi:10.1001/jamanetworkopen.2023.0490)
Supplement: Supplement 2. — Data Sharing Statement [file jamanetwopen-e230490-s002.pdf]

Data Sharing Statement

Goldstein. Multinational Analysis of Estimated Health Care Costs Related to Extended-Interval Fixed Dosing of Checkpoint Inhibitors. *JAMA Netw Open*. Published February 23, 2023. doi:10.1001/jamanetworkopen.2023.0490

Data
Data available: No

Additional Information
Explanation for why data not available: there is no additional patient data - everything is included in the manuscript and supplemental material.
